# Genomic ancestry estimation quantifies use of wild species in grape breeding

**DOI:** 10.1186/s12864-016-2834-8

**Published:** 2016-06-30

**Authors:** Zoë Migicovsky, Jason Sawler, Daniel Money, Rudolph Eibach, Allison J. Miller, James J. Luby, Andrew R. Jamieson, Dianne Velasco, Sven von Kintzel, John Warner, Walter Wührer, Patrick J. Brown, Sean Myles

**Affiliations:** Department of Plant and Animal Sciences, Faculty of Agriculture, Dalhousie University, Truro, NS Canada; Anandia Labs, 2259 Lower Mall, Vancouver, BC Canada; JKI Institute for Grapevine Breeding, Geilweilerhof, Germany; Department of Biology, Saint Louis University, Saint Louis, MO USA; Department of Horticultural Science, University of Minnesota, St. Paul, MN USA; Agriculture & Agri-Food Canada, Atlantic Food & Horticulture Research Centre, Kentville, NS Canada; USDA-ARS, National Clonal Germplasm Rep, Davis, CA USA; Jost Vineyards, 48 Vintage Lane, Malagash, NS Canada; Warner Vineyards, 391 Thorpe Rd, RR#2, Centreville, NS Canada; Wührer Vineyards, 187 Highway 221, RR#1, Kingston, NS Canada; Department of Crop Sciences, University of Illinois, Urbana, IL USA

## Abstract

**Background:**

Grapes are one of the world’s most valuable crops and most are made into wine. Grapes belong to the genus *Vitis,* which includes over 60 inter-fertile species. The most common grape cultivars derive their entire ancestry from the species *Vitis vinifera,* but wild relatives have also been exploited to create hybrid cultivars, often with increased disease resistance.

**Results:**

We evaluate the genetic ancestry of some of the most widely grown commercial hybrids from North America and Europe. Using genotyping-by-sequencing (GBS), we generated 2482 SNPs and 56 indels from 7 wild *Vitis*, 7 *V. vinifera,* and 64 hybrid cultivars. We used a principal component analysis (PCA) based ancestry estimation procedure and verified its accuracy with both empirical and simulated data. *V. vinifera* ancestry ranged from 11 % to 76 % across hybrids studied. Approximately one third (22/64) of the hybrids have ancestry estimates consistent with F1 hybridization: they derive half of their ancestry from wild *Vitis* and half from *V. vinifera*.

**Conclusions:**

Our results suggest that hybrid grape breeding is in its infancy. The distribution of *V. vinifera* ancestry across hybrids also suggests that backcrosses to wild *Vitis* species have been more frequent than backcrosses to *V. vinifera* during hybrid grape breeding. This pattern is unusual in crop breeding, as it is most common to repeatedly backcross to elite, or domesticated, germplasm. We anticipate our method can be extended to facilitate marker-assisted selection in order to introgress beneficial wild *Vitis* traits, while allowing for offspring with the highest *V. vinifera* content to be selected at the seedling stage.

**Electronic supplementary material:**

The online version of this article (doi:10.1186/s12864-016-2834-8) contains supplementary material, which is available to authorized users.

## Background

Grapes are one of the world’s most valuable crops and although grown primarily for wine, they are also used fresh, dried and in juice [1]. In 2013, grapes had the 2^nd^ highest global gross production value among fruit crops, exceeded only by tomato [2]. Grapes belong to the genus *Vitis*, which includes over 60 inter-fertile species spread broadly across the northern hemisphere [3]. However, based on total global area in 2010, over 98 % of wine grapes belong to a single species, *Vitis vinifera* [4]. Almost all grape cultivars grown commercially are either *V. vinifera* or hybrids that include *V. vinifera* parentage [1].

In addition to the use of one *Vitis* species for almost all grape growing, grapes are predominately grown using vegetative propagation, which has resulted in extensive clonal relationships and limited diversity. The wine industry’s preference for traditional varieties makes the acceptance of new *V. vinifera* cultivars difficult [5, 6]. A study by Myles et al. [7] found 58 % of the 950 grape cultivars examined had at least one clonal relationship. Among the unique cultivars, 74.8 % had a first-degree relationship with at least one other cultivar. This extensive inter-relatedness and lack of diversity have left grape cultivars susceptible to many continually evolving pathogens [7, 8]. For example, Pierce’s disease currently costs the California wine industry approximately $92 million annually [9]. The future of the wine industry relies on the exploration of new genetic diversity through breeding.

Crop wild relatives (CWRs) provide a useful source of genetic variation for crop improvement [10–12]. An overview of 19 different crops found that more than 80 % of beneficial traits from CWR genes were involved in pest and disease resistance [12]. By 1997, genomic crop improvements made due to CWRs had an estimated global benefit of $115 billion annually [13]. Due to disease susceptibility of *V. vinifera* cultivars, settlers to North America had great difficulty growing the vine. These early settlers grew native, wild vines such as *V. labrusca* and *V. aestivalis* and hybridized them with *V. vinifera* [14]. Significant exploitation of CWRs began in the 1850s when the phylloxera louse devastated European vineyards. Breeders used American wild *Vitis* species to develop rootstocks resistant to phylloxera*,* rescuing the wine industry. Commercial *V. vinifera* wine cultivars are still grafted onto these phylloxera-resistant rootstocks [6, 15].

Largely in response to the phylloxera crisis, wild *Vitis* were also used in scion breeding. However, the initial hybrids were generally considered undesirable for wine production due to unfavorable aromas and tastes inherited from the wild *Vitis* parents [16–19]. Sustained breeding enabled the development of hybrid cultivars with improved disease resistance and without the undesirable flavor compounds, including German varieties such as ‘Phoenix’ and ‘Orion’ [6]. Early French breeders, including Eugene Kuhlmann and Pierre Castel, also created well-known hybrids such as ‘Marechal Foch’ and ‘Castel’. However, despite the promise of novel hybrid grape cultivars, their use was met with strong resistance. France introduced several wine “quality laws” prohibiting the use of many French-American hybrids [1, 20]. French regulations influenced the perception of hybrid grape cultivars, as well as the European Union wine classification, which outlawed hybrids from the highest quality level [20].

Although it is widely believed that nearly all commercial grape varieties derive their entire ancestry from *V. vinifera*, there is increasing evidence that wild *Vitis* species may have been incorporated more often than previously assumed. Estimates of *V. vinifera* ancestry frequently rely on historical pedigrees from breeders, but these records may be flawed. Genomics provides a powerful tool for detecting pedigree errors and wild *Vitis* ancestry. For example, a recent study used nuclear microsatellite markers to determine that 33 % of the 381 breeder pedigrees examined were incorrect. In most cases, the paternal parent was incorrectly identified, likely due to pollen contamination [21]. Most recently, a genomic analysis uncovered that the most important Japanese wine cultivar, ‘Koshu’, contained 30 % wild ancestry despite being commonly classified as entirely *V. vinifera* [22].

In addition to illuminating the contribution of wild *Vitis* to commercial grapes, genomics can help breeders introgress desirable traits from wild relatives into new grape cultivars. Marker-assisted selection (MAS) uses genetic markers either responsible for a phenotype or strongly linked to it. MAS is especially helpful in long-lived perennial crops, like grapes, where selection can be made at the seed or seedling stage, eliminating the time and money required for the plant to fully mature [23]. Moreover, combining markers linked to key traits with genomic ancestry estimates can enable breeders to select the progeny with the highest *V. vinifera* content as well as the desirable trait from the wild relative.

To enable genomics-assisted ancestry estimation in grapes, Sawler et al. [24] estimated *V. vinifera* ancestry in interspecific *Vitis* hybrids using single nucleotide polymorphism (SNP) array data from 127 accessions in the grape germplasm collection of the United States Department of Agriculture (USDA). However, the USDA collection contains relatively few commonly grown commercial hybrid cultivars. To gain insight into the ancestry across the most common commercial hybrids, we generated genotyping-by-sequencing (GBS) data and quantified *V. vinifera* ancestry from 64 of the most widely grown commercial hybrids from North America and Europe. We find that *V. vinifera* ancestry ranged from 11 % to 76 % across our sample of hybrid varieties. The distribution of ancestry across hybrids suggests the unusual practice of breeders backcrossing more frequently to wild *Vitis* species than to *V. vinifera* during hybrid grape breeding.

## Methods

### Sample collection and genotype calling

Leaf tissue was collected from 63 commercial grape varieties from Canada (Nova Scotia), Germany and the United States. We also used samples from 11 *V. vinifera*, 6 hybrids, and 15 wild accessions from the USDA grape germplasm collection which were previously genotyped in Sawler et al. [24]. DNA was extracted using commercial extraction kits. A list of all samples is available in Additional file 1: Table S1.

A single GBS library from 96 samples was generated according to Elshire et al. [25] using two different pairs of restriction enzymes (HindIII-HF/BfaI, HindIII-HF/MseI) and was sequenced using Illumina Hi-Seq 2000 technology. Reads were aligned to the 12X grape reference genome from GENOSCOPE (http://www.genoscope.cns.fr/externe/GenomeBrowser/Vitis/) using the Tassel/BWA version 5 pipeline with minimum quality score (mnQS) of 20 and minimum kmer count (c) of 3 to generate a genotype matrix with 830,822 sites [26, 27]. All other default parameters were used.

### Data curation

VCFtools v0.1.12b [28] was used to filter for biallelic sites as well as a minimum number of reads (minDP) of 8. The file was converted into PLINK format and SNPs with <20 % missing data were retained using PLINK v1.07 [29, 30]. Accessions with >20 % missing data were removed, followed by SNPs with a minor allele frequency (MAF) <0.05. SNPs with excess heterozygosity (i.e. failed a Hardy-Weinberg equilibrium test with a *p*-value < 0.001) were also removed, resulting in 80 accessions and 6664 sites remaining. An identity-by-state (IBS) similarity matrix was calculated using PLINK for hybrid samples (Additional file 2: Table S2). Missing genotypes were imputed using LinkImpute [31] with optimized values of 6 and 17 for parameters *l* and *k*, respectively, which resulted in an estimated genotype imputation accuracy of 91 %.

In order to perform a PCA-based admixture analysis, equal ancestral sample sizes are required [32]. We removed two random *V. vinifera* samples, and the resulting dataset contained 78 samples, which included ancestral populations of 7 wild *Vitis* samples and 7 *V. vinifera*. Only SNPs with MAF >0.1 in the ancestral populations were retained across all samples.

We pruned for linkage disequilibrium using PLINK by considering a window of 10 SNPs, removing one SNP from a pair if r^2^ was >0.5 then shifting the window by 3 SNPs and repeating the procedure (PLINK command: indep-pairwise 10 3 0.5). 2538 sites, which included 56 indels and 2482 SNPs, remained for principal component analysis (PCA). The median distance between SNPs remaining after filtering was 1086 bp and the inter-SNP distribution can be seen in Additional file 3: Figure S1.

### Ancestry estimation

**Fig. 1:**
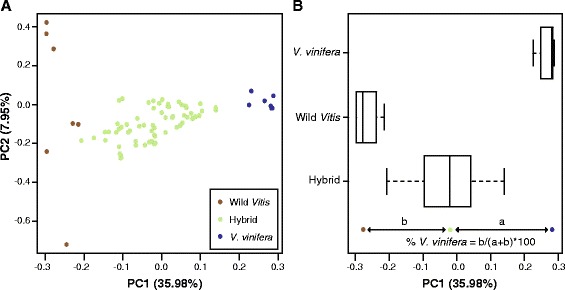
PCA-based ancestry estimation using 2482 SNPs and 56 indels for 7 wild *Vitis*, 7 *V. vinifera,* and 64 hybrid samples. **a** PCs were generated using wild *Vitis* and *V. vinifera* samples. The proportion of the variance explained by each PC is shown in parentheses along each axis. Hybrids were projected onto the axes. **b** Boxplots of PC1 values for wild *Vitis*, *V. vinifera*, and hybrid cultivars as well as a visual description of the calculation used for ancestry estimation. Further details are found in the Methods

We calculated principal component (PC) axes using the ancestral *V. vinifera* and wild *Vitis* samples and then projected hybrid cultivars onto these axes using smartpca from the EIGENSOFT v.6.0.4 software package (Fig. 1a) [33, 34]. Based on PCA projection, ancestry coefficients for each hybrid were estimated using a similar approach described in [24, 35]. The Euclidean distance between a particular hybrid cultivar and the mean value for *V. vinifera* (a) and wild *Vitis* (b) populations along PC1 was calculated and the percentage of *V. vinifera* was determined using the formula ‘% *V. vinifera* = b/(a + b)*100’ (Fig. 1b).

### Simulations of admixture

In order to determine the accuracy of the PCA-based ancestry estimates, we generated simulated offspring using data from the genotyped samples as described in Sawler et al. [24]. We estimated the proportion *V. vinifera* ancestry from simulated F1 hybrids, F1 x *V. vinifera* backcrosses and F1 x wild *Vitis* backcrosses, which are expected to have 50 %, 75 % and 25 % *V. vinifera* ancestry, respectively. For the F1 hybrids, a parent was randomly selected from each two ancestral populations, and parental genotypes were combined by randomly sampling one allele at each site. Linkage disequilibrium between sites was ignored and the process was repeated 10,000 times in order to generate 10,000 F1 offspring. The procedure was repeated with a randomly chosen simulated F1 as one parent and a randomly chosen wild *Vitis* (*n* = 10,000) or *V. vinifera* (*n* = 10,000) as the other, in order to simulate backcrossing to the ancestral populations. The percentage *V. vinifera* ancestry and 95 % confidence interval were calculated for all simulated populations.

## Results and discussion

### Method verification

Wild *Vitis* species can be used in grape breeding programs to introgress disease and abiotic stress resistance into susceptible germplasm belonging to the domesticated grape, *V. vinifera*. Commercial cultivars with wild *Vitis* ancestry are often referred to as “hybrids”. An evaluation of ancestry across commercial hybrids can provide insight into the history of hybrid grape breeding and a foundation for future efforts to select for ancestry based on marker data. Previous work provided accurate ancestry estimates of interspecific grape cultivars using Vitis9KSNP array data for cultivars belonging to the USDA germplasm collection [24]. We applied the same PCA-based method to evaluate the ancestry of some of the most widely grown hybrid cultivars sampled from North America and Europe using GBS data.

PCA provides a clear separation of wild *Vitis* and *V. vinifera* samples along PC1, with commercial hybrids found between the two ancestral groups (Fig. 1a). The projected position of a hybrid along PC1 was used to calculate its percentage *V. vinifera* ancestry (Fig. 1b).

**Fig. 2:**
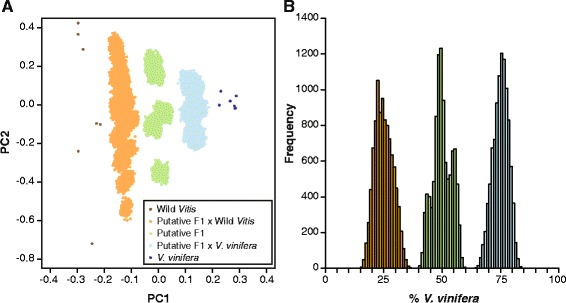
Simulation of hybrids (10,000 of each). **a** Simulated hybrids including F1 hybrids, F1 backcrossed to *V. vinifera* and F1 backcrossed to wild *Vitis* were projected onto axes generated using wild *Vitis* and *V. vinifera* samples **b** Distribution of ancestry estimates for simulated populations

In order to evaluate the accuracy of our ancestry estimates, we performed *in silico* crosses between wild *Vitis* and *V. vinifera* populations using our genome-wide SNP data to simulate F1 hybrids as well as hybrids generated from F1 simulated hybrids backcrossed to *V. vinifera* or wild *Vitis*. The simulated progeny were projected onto PC axes determined using the ancestral populations and the resulting PCA plot is shown in Fig. 2a.

The expected *V. vinifera* content in an F1 offspring with one *V. vinifera* and one wild *Vitis* parent is 50 %, and the mean estimated content in the simulated F1 population described here was 50.1 %, with a 95 % confidence interval (CI) ranging from 42.7 % to 57.2 %. In progeny produced by an F1 hybrid backcrossed to wild *Vitis*, the expected *V. vinifera* content is 25 %, which was the mean estimate of our simulated data, with a 95 % CI of 18.4 % to 32.6 %. Finally, the mean *V. vinifera* content in simulated F1 hybrids backcrossed to *V. vinifera* is expected to be 75 %, and our results have a mean value of 75.1 %, with a 95 % CI of 68.5 % to 80.9 %. The proximity of our simulated values to expected values provides support for the accuracy of our method, but it is worth noting that our 95 % confidence intervals indicate that estimates may deviate by as much as 7–8 % from the expected value. Moreover, the accuracy of our estimates may decrease in cases where crosses are generated from parents whose ancestry differs significantly from the samples used as ancestral populations in the present study. Ancestry estimates for simulated progeny are shown in Fig. 2b.

### Commercial grape ancestry estimation

**Fig. 3:**
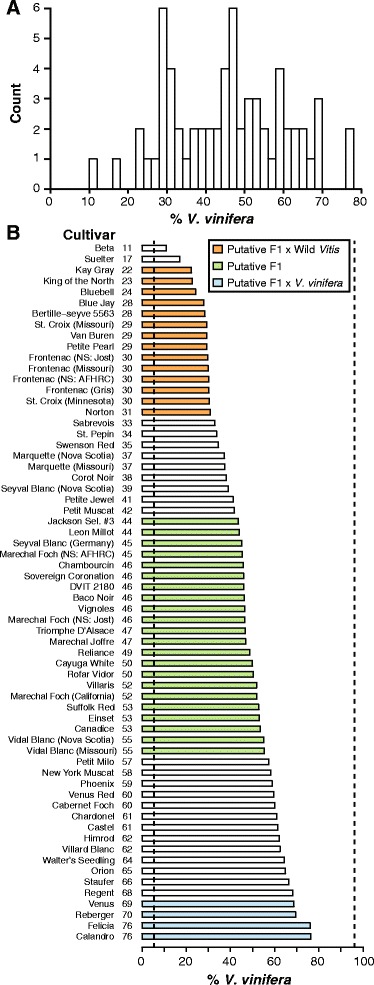
Estimated *V. vinifera* content in 64 commercial grape hybrids. Estimates are based on 2538 sites. **a** Distribution of *V. vinifera* ancestry estimates in hybrids (**b**) *V. vinifera* ancestry estimates for each cultivar. Bars are colored if a hybrid cultivar’s ancestry estimate falls within the 95 % confidence interval of a F1, F1 x wild *Vitis*, or F1 x *V. vinifera* cross, based on simulated values. Dotted lines indicate mean values for the wild *Vitis* and *V. vinifera* samples

The distribution of *V. vinifera* content estimated for the hybrid grape cultivars examined in this work is found in Fig. 3a, and the ancestry estimates for each cultivar are listed in Fig. 3b.

Hybrids previously genotyped in Sawler et al. [24] and replicated in this study using GBS include ‘Bertille-seyve 5563’ (DVIT 169), ‘Van Buren’ (DVIT 1129), ‘Rofar Vidor’ (DVIT 2258), DVIT 2180, ‘Jackson Sel. #3’ (DVIT 2916), and ‘Marechal Foch’ (California) (DVIT 214). The ancestry estimates for these samples differed by 2–5 % from those previously estimated, with the exception of DVIT 2180 where our estimate of *V. vinifera* ancestry was 19 % higher than in the previous work. DVIT 2180 is an unnamed accession simply identified as a *Vitis* species by the USDA. Given that the tissue for both studies was collected separately, the large difference in our estimates may be due to mislabelling or sample mix-up. Regardless of this discrepancy, the position of this sample in PC space confirms that it is indeed a hybrid sample (Fig. 1a).

In order to further confirm the accuracy of our ancestry estimates, we compared *V. vinifera* ancestries inferred from well-known pedigrees to our genomics-based ancestry estimates. For example, ‘Beta’ is a cross between *Vitis riparia* and ‘Concord’, a *Vitis labrusca* cross thought to possess some *V. vinifera* ancestry due in part to its hermaphroditic flowers [36, 37]. Sawler et al. [24] estimated the *V. vinifera* content of ‘Concord’ as 31 %. Based on these values, the percentage *V. vinifera* found in ‘Beta’ is expected to be approximately 16 %, and it was estimated as 11 % here (Fig. 3a). ‘Baco Noir’ is a known F1 hybrid between ‘Folle Blanc’ (*V. vinifera*) and *V. riparia*, and therefore it is expected to be 50 % *V. vinifera.* Our estimate is 46 %, which falls within the 95 % confidence interval of the *V. vinifera* ancestry estimates from our simulated F1 hybrid offspring. In these two cases, our genomics-based ancestry estimates are consistent with pedigree-based estimates.

Our study also included several cultivars collected from multiple locations, and the ancestry estimates were generally similar or equivalent for these replicates from different geographic regions. For example, ‘Frontenac’ sampled from two locations in Nova Scotia, Missouri, as well as a Gris sport, were all estimated to be 30 % *V. vinifera.* ‘Marquette’ samples from both Nova Scotia and Missouri were estimated to contain 37 % *V. vinifera*. However, the ancestry estimate (52 %) for a ‘Marechal Foch’ accession retrieved from the USDA germplasm collection was 6 % and 7 % higher than the samples collected from two different locations in Nova Scotia. IBS values indicate that this sample is likely not the same cultivar as the ‘Marechal Foch’ grown in Nova Scotia (Additional file 4: Figure S2). Still, all ancestry estimates of ‘Marechal Foch’ fall within the putative F1 range, which is expected given ‘Marechal Foch’ is the offspring of ‘101–14 Mgt.’ (*V. riparia* x *V. rupestris*) x ‘Goldriesling’ (*V. vinifera*). ‘Leon Millot’ (44 %) and ‘Marechal Joffre’ (47 %) are siblings of ‘Marechal Foch’, and their ancestry estimates also fall within the range expected from an F1 hybrid (Fig. 3b) [38].

Within cultivar differences in ancestry estimates may be due partially to genotyping error. Curation error also leads to the mislabeling of samples and misidentification of cultivars. Previous work on *V. vinifera* cultivars from the USDA collection revealed widespread curation error [7], and recent work on the same collection found that the species names assigned to samples were incorrect in approximately 4 % of cases [24]. In another example, three different Italian varieties all referred to as ‘Bonarda’ had no direct genetic relationship with each other [39]. Thus, curation error represents a likely source for the discrepancies we observe between samples with identical names.

While our data do not allow us to resolve first-degree relationships, we did examine the distribution of IBS values based on expected relationships derived from pedigree data (Additional file 4: Figure S2). We found that, while many cultivars do share alleles in a manner that supports their expected relationship, several pairs of samples that are supposed to be either geographic replicates or first-degree relatives did not have IBS values consistent with their pedigrees. For example, the IBS value for ‘Villaris’ and ‘Felicia’ (0.83) was at least 0.02 lower than all other sibling pairs examined. Additionally, the ‘Seyval Blanc’ sampled from Germany does not resemble the ‘Seyval Blanc’ from Nova Scotia to the degree we expect. In both cases, the *V. vinifera* ancestry estimates also differed. Furthermore, ‘Orion’, ‘Staufer’ and ‘Phoenix’ are all progeny of crosses between ‘Villard Blanc’ (62 %) and *V. vinifera* varieties, which has been confirmed by simple sequence repeat genotyping (Rudolf Eibach, personal communication). However, the expected ancestry for these progeny based on pedigree information should be higher (~81 %) than what we observe (59 %–65 %). Further work is required in order to confirm potential sample mislabeling, cross-contamination, or genotyping error.

### Wild species introgression

Often the best source for improvement of a crop plant is its wild relatives [11]. One crop that has benefited greatly from the use of wild relatives in breeding is tomato. Disease resistance in most commercial tomato cultivars is the result of genes introgressed from wild species [40, 41]. However, recurrent backcrossing to elite varieties is performed for several generations in order to remove undesirable genes introduced from the wild relative [41]. In tomato, it is customary to continue backcrossing to elite germplasm for 4 to 6 generations before the resulting hybrid is tested commercially [42].

In comparison to tomato, grape breeding appears to still be in its infancy. Approximately one third (22/64) of the hybrids analyzed in this study have *V. vinifera* content consistent with F1 hybridization (Fig. 3b). Our results suggest that grape breeders have not extensively backcrossed with *V. vinifera* in order to introgress wild genes of interest. The distribution of *V. vinifera* ancestry across hybrids actually implies that backcrosses to wild *Vitis* species have been more frequent than backcrosses to *V. vinifera* during hybrid grape breeding. Breeders may have generated hybrids with high wild content when aiming to introgress numerous beneficial traits from wild relatives over a small number of generations. Further local ancestry estimates would be required in order to determine the number of generations of crossing.

The high number of hybrids consistent with F1 hybridization suggests that, overall, recent hybrid grape breeding has not followed standard breeding practices that aim to introgress desirable traits from wild species by repeatedly backcrossing to elite germplasm. Alternatively, because breeders often target numerous traits for introgression from the wild, the optimal *V. vinifera* content may be lower than the desired elite content in other crops. Ultimately, the crucial factor will be which desirable parts of each ancestral genome are captured, rather than the final *V. vinifera* percentage.

One instance where repeated backcrossing to *V. vinifera* has been exploited is in the development of Pierce’s disease (PD) resistant wine grapes by tracking PD resistance alleles from the wild species *V. arizonica* through MAS [43]. Seedlings resistant to PD were repeatedly backcrossed to *V. vinifera*, resulting in progeny with 97 % *V. vinifera* ancestry in the fifth generation, a value much higher than any estimates of commercial cultivars examined in this study [44]. There are many more opportunities for desirable traits, such as cold hardiness, to be introgressed from wild *Vitis* species into novel elite cultivars [45].

The use of molecular markers can also allow breeders to introgress multiple resistance genes into a single variety, a process called pyramiding [46]. ‘Regent’ is a cross between ‘Diana’, a *V. vinifera* variety, and the hybrid grape ‘Chambourcin’, which has 46 % *V. vinifera* ancestry according to our work. Based on these values, the expected *V. vinifera* ancestry of ‘Regent’ is approximately 73 %, and our estimate is 68 %. The complex pedigree of ‘Regent’ enabled the introgression of mildews and botrytis disease resistance from several *Vitis* species as well as high frost tolerance and early maturity [47]. In 2013, ‘Regent’ ranked 12^th^ in Germany according to total acreage [48]. Recently, 'Regent' was crossed with VHR 3082-1-42 (*Muscadinia rotundifolia* x *V. vinifera*, then backcrossed four times with *V. vinifera)* to successfully combine powdery and downy mildew resistance genes into a single variety whose ancestry likely exceeds 80 % *V. vinifera* [49].

The Institute for Grapevine Breeding Geilweilerhof, which developed ‘Regent’, bred 6 of the 7 cultivars with the highest *V. vinifera* content in our study (Fig. 3b). Thus, some breeders have produced hybrids with a high percentage of *V. vinifera* ancestry while retaining desirable characteristics from wild species. However, the overall lack of evidence for repeated backcrossing to *V. vinifera* in hybrid grape breeding indicates that grape breeders have yet to fully exploit the potential of combining key traits from wild species into novel cultivars with high *V. vinifera* content.

## Conclusions

By examining the ancestry of 64 commercially grown grape hybrids using PCA-based ancestry estimation, we found that approximately one third of hybrids have ancestry consistent with F1 hybridization: they derive half of their ancestry from wild *Vitis* and half from *V. vinifera*, suggesting that hybrid grape breeding is in its infancy. If backcrossing to *V. vinifera* was more widely adopted, we anticipate increased acceptance of hybrid grape varieties. Improved hybrid cultivars with higher *V. vinifera* ancestry could eventually lead to the relaxation of regulations against planting hybrid grapes, and ultimately a proliferation of grape cultivars with increased abiotic and disease resistance as well as favored wine qualities.

We anticipate our method can be extended to facilitate marker-assisted selection by allowing for offspring with the highest *V. vinifera* content to be selected at the seedling stage. In combination with MAS, ancestry estimates, such as those described here, can enable the continued improvement of grape by exploiting the diversity of wild *Vitis* species while maintaining desirable *V. vinifera* characteristics.

## Abbreviations

CI, confidence interval; CWRs, crop wild relatives; GBS, genotyping-by-sequencing; IBS, identity-by-state; MAF, minor allele frequency; MAS, marker-assisted selection; PC, principal component; PCA, principal component analysis; PD, Pierce’s disease; SNP, single nucleotide polymorphism; USDA, United States Department of Agriculture

## Additional files

Additional file 1: Table S1. A list of the 78 cultivars used in this study as well as location and institute. Sample information is also provided for the additional 16 samples which were discarded during the genotyping analysis pipeline and they are highlighted in yellow. (XLSX 27 kb) Additional file 2: Table S2. Matrix of genome-wide average IBS values for 64 hybrids. (XLSX 82 kb) Additional file 3: Figure S1. Distance (kb) between filtered SNPs used for ancestry estimation. (PDF 96 kb) Additional file 4: Figure S2. Distribution of IBS values for expected replicates (orange), siblings (blue) and parent/offspring (red). (A) Histogram of IBS values calculated in hybrid samples only. Dotted lines are drawn at values for expected first degree relationships as well as replicates. (B) Expected relationships between cultivars with their associated IBS values. (PDF 264 kb)
