# Supplementary figures and images for: Genomic ancestry estimation quantifies use of wild species in grape breeding
Source: BMC Genomics. 2016 Jun 30;17:478. doi: 10.1186/s12864-016-2834-8 (PMC4928267; doi:10.1186/s12864-016-2834-8)

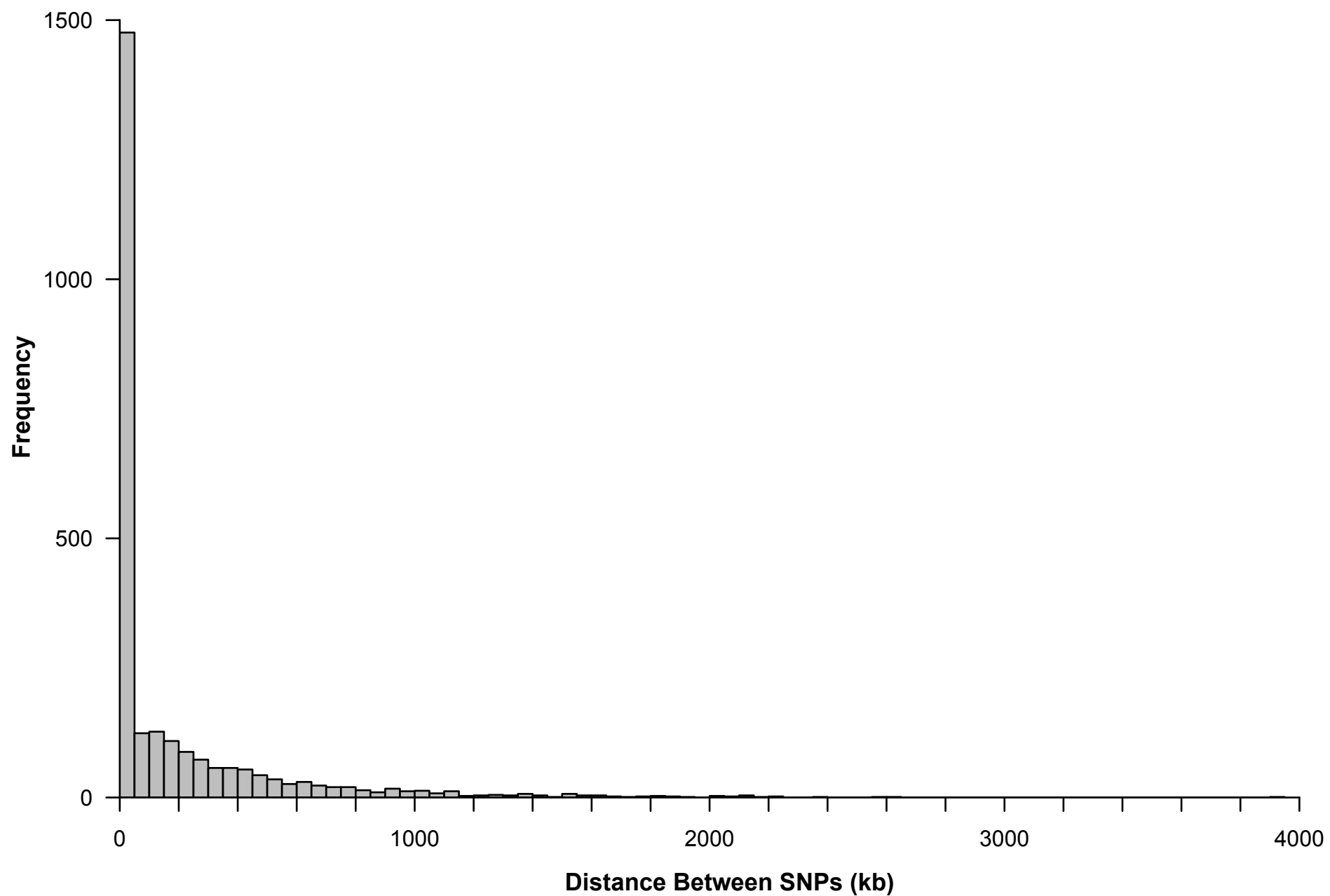

Supplement: Additional file 3: Figure S1. — Distance (kb) between filtered SNPs used for ancestry estimation. (PDF 96 kb) [file 12864_2016_2834_MOESM3_ESM.pdf]
