# Supplementary material for: Genomic ancestry estimation quantifies use of wild species in grape breeding
Source: BMC Genomics. 2016 Jun 30;17:478. doi: 10.1186/s12864-016-2834-8 (PMC4928267; doi:10.1186/s12864-016-2834-8)
Supplement: Additional file 4: Figure S2. — Distribution of IBS values for expected replicates (orange), siblings (blue) and parent/offspring (red). (A) Histogram of IBS values calculated in hybrid samples only. Dotted lines are drawn at values for expected first degree relationships as well as replicates. (B) Expected relationships between cultivars with their associated IBS values. (PDF 264 kb) [file 12864_2016_2834_MOESM4_ESM.pdf]

A

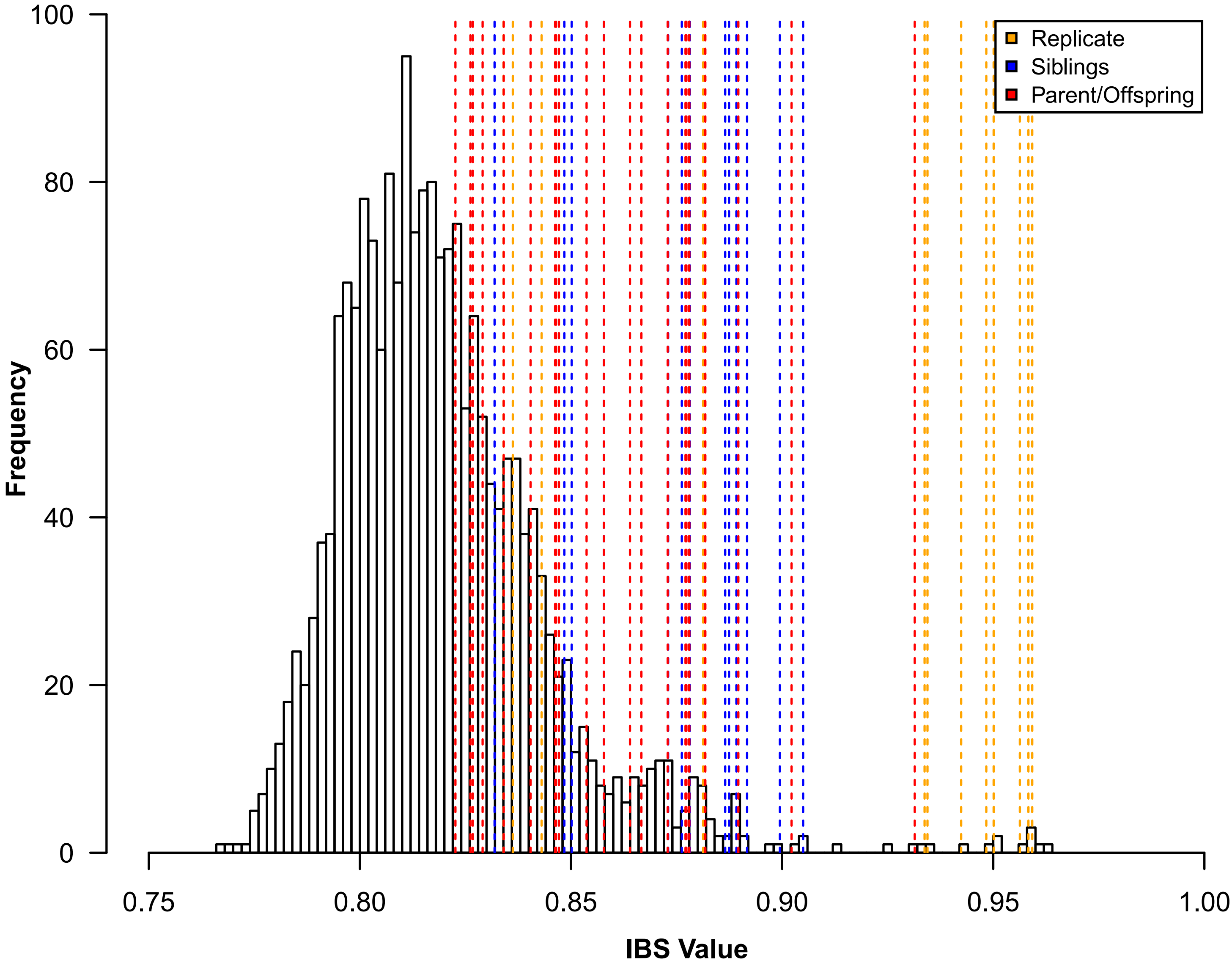

B

Cultivar 1	Cultivar 2	Relationship	IBS Value
Felicia	Vidal Blanc (Missouri)	Parent/Offspring	0.82
Cayuga	Seyval Blanc (Germany)	Parent/Offspring	0.83
Felicia	Vidal Blanc (Nova Scotia)	Parent/Offspring	0.83
Chardonel	Seyval Blanc (Germany)	Parent/Offspring	0.83
Villaris	Felicia	Sibling	0.83
Seyval Blanc (Nova Scotia)	Seyval Blanc (Germany)	Replicate	0.83
Cabernet Foch	Marechal Foch (California)	Parent/Offspring	0.83
Marechal Foch (Nova Scotia: Jost)	Marechal Foch (California)	Replicate	0.84
Phoenix	Villard Blanc	Parent/Offspring	0.84
Marechal Foch (Nova Scotia: AFHRC)	Marechal Foch (California)	Replicate	0.84
Beta	Bluebell	Parent/Offspring	0.85
Orion	Villard Blanc	Parent/Offspring	0.85
Staufer	Villard Blanc	Parent/Offspring	0.85
Leon Millot	Marechal Foch (California)	Sibling	0.85
Marechal Foch (California)	Marechal Joffre	Sibling	0.85
Beta	Blue Jay	Parent/Offspring	0.85
Beta	Suelter	Sibling	0.86
Cabernet Foch	Marechal Foch (Nova Scotia: Jost)	Parent/Offspring	0.86
Cabernet Foch	Marechal Foch (Nova Scotia: AFHRC)	Parent/Offspring	0.86
Reliance	Suffolk Grape	Parent/Offspring	0.87
Regent	Chambourcin	Parent/Offspring	0.87
Sabrevois	St. Croix (Minnesota)	Sibling	0.87
Phoenix	Orion	Sibling	0.88
Vidal Blanc (Missouri)	Villaris	Parent/Offspring	0.88
Vidal Blanc (Nova Scotia)	Villaris	Parent/Offspring	0.88
Petite Jewel	Canadice	Parent/Offspring	0.88
Cayuga	Seyval Blanc (Nova Scotia)	Parent/Offspring	0.88
Phoenix	Staufer	Sibling	0.88
Sabrevois	St. Croix (Missouri)	Sibling	0.88
Marechal Foch (Nova Scotia: AFHRC)	Marechal Foch (Nova Scotia: Jost)	Replicate	0.88
Himrod	Canadice	Parent/Offspring	0.88
Chardonel	Seyval Blanc (Nova Scotia)	Parent/Offspring	0.88
Orion	Staufer	Sibling	0.89
Leon Millot	Marechal Foch (Nova Scotia: Jost)	Sibling	0.89
Leon Millot	Marechal Foch (Nova Scotia: AFHRC)	Sibling	0.89
Sovereign Coronation	Himrod	Parent/Offspring	0.89
Marechal Foch (Nova Scotia: Jost)	Marechal Joffre	Sibling	0.89
Marechal Foch (Nova Scotia: AFHRC)	Marechal Joffre	Sibling	0.90
Leon Millot	Marechal Joffre	Sibling	0.90
Regent	Calandro	Parent/Offspring	0.91
Regent	Reberger	Parent/Offspring	0.93
St. Croix (Missouri)	St. Croix (Minnesota)	Replicate	0.93
Marquette (Missouri)	Marquette (Nova Scotia)	Replicate	0.93
Vidal Blanc (Nova Scotia)	Vidal Blanc (Missouri)	Replicate	0.94
Frontenac (Gris)	Frontenac (Missouri)	Replicate	0.95
Frontenac (Gris)	Frontenac (Nova Scotia: Jost)	Replicate	0.95
Frontenac (Nova Scotia: AFHRC)	Frontenac (Gris)	Replicate	0.95
Frontenac (Nova Scotia: AFHRC)	Frontenac (Missouri)	Replicate	0.96
Frontenac (Missouri)	Frontenac (Nova Scotia: Jost)	Replicate	0.96
Frontenac (Nova Scoia: AFHRC)	Frontenac (Nova Scotia: Jost)	Replicate	0.96
